# Designing a combined liothyronine (LT3), L- thyroxine (LT4) trial in symptomatic hypothyroid subjects on LT4 - the importance of patient selection, choice of LT3 and trial design

**DOI:** 10.3389/fendo.2023.1282608

**Published:** 2023-11-16

**Authors:** Lakdasa D. Premawardhana, Peter Nicholas Taylor, Onyebuchi E. Okosieme, Mohamed A. Adlan, Emmanuel K. Obuobie, Colin Mark Dayan

**Affiliations:** ^1^ Thyroid Research Group, Division of Infection and Immunity, Cardiff University School of Medicine, Heath Park, Cardiff, United Kingdom; ^2^ Section of Endocrinology, Ysbyty Ystrad Fawr and Royal Gwent Hospitals, Aneurin Bevan University Health Board, Newport, United Kingdom

**Keywords:** hypothyroidism, combined LT3 and LT4 treatment, deiodinase 2 polymorphism, randomized controlled trial, poor quality of life (QoL)

## Abstract

Approximately 10%–15% of subjects with hypothyroidism on L-thyroxine (LT4) alone have persistent symptoms affecting their quality of life (QoL). Although the cause is unclear, there is evidence that “tissue T3 lack” may be responsible. If so, combining liothyronine (LT3) with LT4 would be helpful. However, randomized controlled trials (RCT), have not established greater efficacy for the LT3 + LT4 combination in these subjects than for LT4 alone. While the trial design may have been responsible, the use of unphysiological, short-acting LT3 preparations and non-thyroid-specific patient-reported outcome measures (PROMs) may have contributed. We recommend attention to the following aspects of trial design for future RCTs of LT3 + LT4 compared to LT4 alone: (a) Subject selection—(i) measurable symptoms (disadvantages should be recognized); (ii) using a validated thyroid specific PROM such as ThyPRO39 or the Composite scale derived from it; (iii) those taking over 1.2 μg/day or 100 μg/day (for pragmatic reasons) of LT4 defining a population likely without intrinsic thyroid activity who depend on exogenous LT4; (iv) recruiting a preponderance of subjects with autoimmune thyroiditis increasing generalisability; and (v) those with a high symptom load with a greater response to combination therapy e.g. those with the deiodinase 2 polymorphism. (b) The use of physiological LT3 preparations producing pharmacokinetic similarities to T3 profiles in unaffected subjects: two long-acting LT3 preparations are currently available and must be tested in phase 2b/3 RCTs. (c) The superiority of a crossover design in limiting numbers and costs while maintaining statistical power and ensuring that all subjects experienced the investigative medication.

## Introduction

Eighteen randomized controlled trials (RCT) of combined liothyronine (LT3) and L-thyroxine (LT4) therapy (LT3 + LT4) have been inconclusive regarding their superiority in improving symptoms and quality of life (QoL) in hypothyroid subjects dissatisfied with LT4 alone (1) (**Table 1**). While 16 RCTs used short acting LT3 preparations in subjects with primary hypothyroidism (autoimmune, post-radioiodine, post-thyroidectomy), one used natural desiccated thyroid (NDT) and one investigated patients with central hypothyroidism (2, 3). This group of “dissatisfied” hypothyroid subjects on LT4 monotherapy constitutes a significant number estimated to be 10%–15% of all hypothyroid patients in the UK (4–6). Therefore, the unmet needs are significant. Their number will increase because of (a) the rising prevalence of hypothyroidism partly accounted for by lower thresholds for starting LT4 in an aging population (7, 8)—prevalence in the USA increased from 9.5% (2012) to 11.7% (2019), with over 78% receiving LT4 alone (9), and (b) a significant proportion of endocrinologists (51%) who are willing to consider prescribing LT3 for these patients (10).

**Table 1:** Clinical trials of LT3 + LT4 combination therapy.

Author (year)	Country	Design	Duration	N (% women)	Dose	Diagnosis
1.Bunevicius (2000)	Lithuania	Crossover	5 W	26 (100)	o.d.	AITD + ThyCa *
2. Bunevicius (2002)	Lithuania	Crossover	5 W	13 (100)	o.d.	Subtotal Thy
3. Clyde (2003)	USA	Parallel	4 M	46 (82)	b.d.	AITD+post ablative
4. Sawka (2003)	USA	Parallel	15 W	40 (90)	b.d.	AITD
5. Walsh (2003)	Australia	Crossover	10 W	110 (93)	o.d.	AITD + post ablative
6. Siegmund (2004)	Germany	Crossover	12 W	26	ND	AITD
7. Appelhof (2005)	Croatia	Parallel	15 W	141 (85)	b.d.	AITD
8. Esco-Morreale (2005)	Spain	Crossover	8 W	28 (100)	o.d.	AITD + post ablative
9. Rodriguez (2005)	USA	Crossover	6 W	30 (89)	o.d.	AITD + post ablative
10. Fadeyev (2005)	Russia	Parallel	6 M	58 (100)	o.d.	AITD
11. Saravanan (2005)	UK	Parallel	12 M	584 (84)	o.d.	ND
*12. Slawik (2007)*	*Germany*	*Crossover*	*5 W*	*32 (8)*	*o.d.*	*Central hypo*
13. Nygaard (2009)	Denmark	Crossover	12 W	68 (93)	o.d.	AITD
14. Valizadeh (2009)	Iran	Parallel	16 W	71 (80)	b.d.	AITD + post ablative
15. Fadeyev (2010)	Russia	Parallel	24 W	36 (100)	o.d.	AITD
*16. Hoang (2013)*	*USA*	*Crossover*	*22 W*	*78 (75)*	*o.d.*	*NDT*
17. Kaminski (2016)	Brazil	Crossover	8 W	32 (94)	o.d.	AITD
18. Krysiak (2018)	Poland	Quasi Rand	24 W	39 (100)	ND	Partial Thyroidectomy

All the above trials included subjects with autoimmune thyroid disease (AITD) except for 12 (Slawik) which recruited subjects with central hypothyroidism and 16 (Hoang) which used NDT (natural desiccated thyroid) as the interventional medication. *Trial contained thyroid cancer patients.

These patients have many symptoms (fatigue, low energy and mood, memory deficits, “brain fog”) and physiological abnormalities (high BMI, abnormal body composition, low resting energy expenditure [REE]), which significantly affect their QoL. Hypothyroid subjects on long-term LT4 alone have (a) a 15% excess of psychological caseness (5), a 5% increase in BMI, increased cholesterol and a 25%–30% increase in cardiovascular and all-cause mortality (10–13), and (b) unphysiological T4/T3 ratios with higher T4 than in the normal population (12, 14). They have many futile consultations and investigations, leading to dissatisfaction with hypothyroidism management. Some of these investigations pertain to excluding other coexisting causes of their symptoms and should be pursued.

The possible causes of persistent symptoms in these patients have been reviewed elsewhere (15, 16). Of these, there is now evidence from animal experiments, population studies, and human experiments to support the “low tissue T3” hypothesis in some (17, 18). It is plausible that this is caused by exogenous LT4 induced inhibition of deiodinase 2 expression (D2, the main enzyme converting intracellular “prohormone” T4 to “active” T3) with differential effects on D2 activity in hypothalamo-pituitary tissues (D2 largely unaffected by excess T4) and peripheral tissues (D2 expression suppressed) (19). These differences in D2 activity normalize serum TSH (the “gold standard” for LT4 dosing), whereas peripheral tissues remain unreplenished with T3 converted from the same LT4. It would therefore seem logical to combine LT3 with LT4 since “tissue T3 lack” may account for their persistent symptoms and poor QoL.

## Designing a RCT in hypothyroid subjects on LT4 with persistent symptoms

The primary objective of a high-quality blinded RCT investigating LT3 + LT4 therapy in hypothyroidism is to provide phase 2b efficacy data showing that LT3 + LT4 is superior to LT4 alone in improving persistent symptoms and QoL while restoring physiological thyroid hormone profiles. The use of patient-reported outcome measures (PROMs) as the primary outcome in RCTs is now internationally validated and accepted (20, 21). Data on pharmacokinetics, safety, and secondary outcomes [weight/BMI, body composition, REE, metabolomic profiles, and bone turnover] should also be sought.

Possible reasons for the inconclusive results of previous RCTs have been discussed in detail elsewhere (22, 23). Here, we highlight the important aspects of designing a future RCT that may provide answers to the disputed question of the efficacy of LT3 + LT4 in this situation:

(1) **Selection of trial subjects**

(a) *Presence of symptoms and threshold for recruitment*

The primary outcome of any RCT of LT3 + LT4 in hypothyroidism should be their effect on symptoms and QoL, as recommended by expert bodies (22, 23). Identifiable and measurable symptoms “quantifying” an unsatisfactory response to LT4 alone should be present at recruitment despite TSH being within the reference range (22, 24).

This is achieved by the following:

(i) The use of thyroid-specific PROMs: Most studies hitherto have used generic, non-thyroid-specific PROMs such as SF-36, HADS, BDI, and GHQ. They are not adequately responsive to changes in symptoms and QoL in patients with benign thyroid diseases (25). There is now a consensus that thyroid specific PROMs, ThyPRO and ThyPRO39 (a shorter version) are the best PROMs for use in RCTs (21, 22, 25). An even shorter version, the Composite Scale (utilizing 22 of 39 ThyPRO39 items), shows comparable validity and reliability (26). These thyroid-specific PROMs fulfil two important criteria: content validity (relevant and acceptable to patients and physicians) and responsiveness (detecting subtle changes over time) (21, 27–29).
  However, patient preference should also be measured as PROMs alone may not accurately capture subtle differences between treatments (21), as it may be affected by the presence/absence of symptoms at recruitment, and thyroid “activity” during the RCT (e.g., LT3 causing iatrogenic hyperthyroidism). One meta-analysis of crossover trials indicated that 48% preferred combination therapy (30), although another suggested that there was no consistent evidence of improved preference, except through chance (31).
(ii) Symptom threshold for recruitment: A ThyPRO Composite scale score of 32 or TSQ >4 has been shown to represent a score that signifies a worse QoL, which is sufficiently above the mean score in patients with hypothyroidism (32). A potential concern about applying a threshold QoL score in recruitment is that individuals with lower scores may have “normalized” their view of their QoL after many years of LT4 treatment, yet still benefit substantially from a change in therapy.
(iii) Quantifying “minimal important change” after treatment: It is important to define the clinical relevance of a change in PROM score in reference to patient perception of benefit. The term “minimal important change” (MIC) is used to define the “smallest change in score in the construct to be measured that patients perceive as important” (33–35). A change of nine points in the ThyPRO Composite score has been identified as a valid MIC (36).

(b) *LT4 dose at recruitment*

Hypothyroid subjects who take a minimum of 1.2 μg/kg/day (21, 22) or 100 μg/day (for pragmatic reasons), have minimal or no endogenous thyroid function and/or derive most of their thyroid hormone from exogenous LT4 therapy. These individuals also have the greatest distrurbance of their circulating T3/T4 ratio (14). They may be more likely to derive benefit from thyroid hormone replacement, including LT3 + LT4, than those who have residual function. This study also excluded many subjects with subclinical hypothyroidism.

(c) *Cause of hypothyroidism*

The only patients without any endogenous thyroid function were those who had undergone total thyroidectomy, e.g., patients with treated thyroid cancer (although such patients on suppressive LT4 therapy should be excluded). However, enrolling only them would considerably reduce the generalizability of the results of an RCT, considerably as most subjects on LT4 have Hashimoto’s thyroiditis. Unrestricted enrolment about the etiology of hypothyroidism and later subgroup analysis is a pragmatic solution.

(d) *The Thr92Ala polymorphism*

Approximately 12%–14% of the UK population is homozygous for a single nucleotide polymorphism (SNP) in the D2 gene (Thr92Ala in its ubiquitination site) (37). The exact mechanism by which the Thr92Ala SNP affects D2 function is unknown because it affects residues distant to the catalytic site, making diminished catalytic activity unlikely. However, it may cause intracellular organelle dysfunction by abnormal translocation to the Golgi apparatus and disruption of mitochondrial function, apoptosis, and growth factor signalling (18, 38). This polymorphism has been shown to be beneficial in the general population (heterozygosity reduces acute lung injury in sepsis by 35% and reduces mortality from COVID-19 by 50%) (39, 40).

The D2 SNP affected symptom load, thyroid hormone levels, and response to LT3 + LT4 in both animal and human studies. It reduces D2 “activity” in the mouse pituitary and HEK-293 cells (38, 41). Mice homozygous for the SNP showed reduced T3 activity in some brain regions associated with behavioral abnormalities (sleep more and reduced physical activity) (38). Methimazole worsened these abnormalities and improved the response to LT3 + LT4.

As expected, human studies have produced significant but occasionally conflicting results: (i) A UK study showed impaired psychological well-being (GHQ) on LT4 alone in homozygotes and a greater response to LT3 + LT4 compared to those with the wild-type allele (37). (ii) A more recent study showed a preference for LT3 + LT4 in patients with either or both monocarboxylase transporter 10 and Thr92Ala-D2 polymorphisms (42). (iii) A study of thyroidectomized patients with at least one Thr92Ala allele demonstrated lower serum T3 levels and normal TSH concentrations (41). (iv) We observed that in individuals on LT4 alone (n = 573), homozygosity for the Thr92Ala polymorphism was associated with reduced QoL (5). (v) A subsequent study failed to replicate our findings but was potentially underpowered—had fewer subjects on LT4 (n = 364) (43). (vi) We have replicated our findings in the HUNT study in Norway (n = 46,712, n = 1,100 on LT4) (44). The Thr92Ala SNP was present in 13% of the population and was not associated with increased HADS scores in subjects not receiving LT4. HADS was 0.71 points higher (0.39–1.02, p <0.001) in subjects on LT4 overall, and 1.83 points higher (0.93–2.73 p <0.001) in those on LT4 who were homozygous for Thr92Ala compared to individuals not on LT4. Thr92Ala non-homozygous individuals on LT4 were 22% more likely than those not on LT4 to reach the threshold for HADS anxiety, whereas homozygous individuals were 208% more likely.

We believe that initial proof of efficacy should be sought in subjects homozygous for the D2 Thr92Ala SNP, as this group has a 3-fold increased symptom burden. Recruiting subjects with symptoms who have the potential to respond well to combination therapy will reduce the size (and cost) of the study and increase its power. Note, however, that even those non-homozygous for Thr92Ala have a 22% increase in symptoms and may benefit from LT3.

In summary, we justified the selection of subjects with the D2 SNP as follows: (1) Subjects homozygous for Thr92Ala have been shown to have a greater symptom burden on LT4 than non-homozygous individuals in two independent populations. (2) They have been shown to have a greater response to LT4 + LT3 than non-homozygous individuals do. (3) However, we have confirmed that non-homozygous individuals also have an excess of psychological morbidity on LT4 compared to the background population, confirming that all subjects with the SNP on LT4 have the potential to benefit from LT3. (4) Power calculations indicate that studies involving Thr92Ala homozygous subjects allow for a 3-fold smaller study, saving time and money.

(2) **Selecting a physiological LT3 preparation**

In subjects with preserved endogenous thyroid function, serum T3 fluctuations are narrow (45). LT3 preparations that deliver profiles mimicking normal human T3 profiles make it easier to adjust the dose and may enhance safety. Current short-acting LT3 preparations are non-physiological and need to be administered at least two/three times per day to achieve a semblance of a physiological profile.

**Figure 1:**
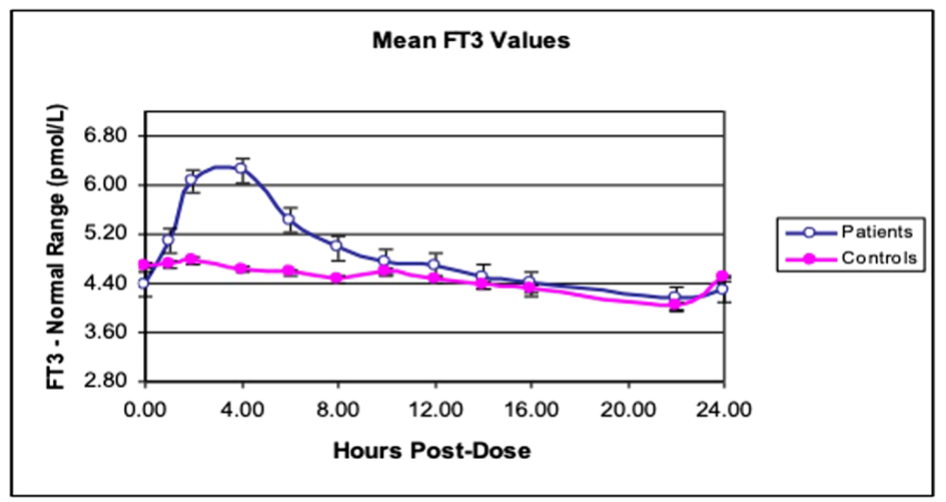
Twenty-four-hour free T3 profiles in hypothyroid subjects. Mean free T3 profiles after giving either LT4 alone (controls) or LT3 + LT4 (patients) in 10 subjects with hypothyroidism. [Reprinted with permission from Saravanan et al. (46)].

**Figure 2:**
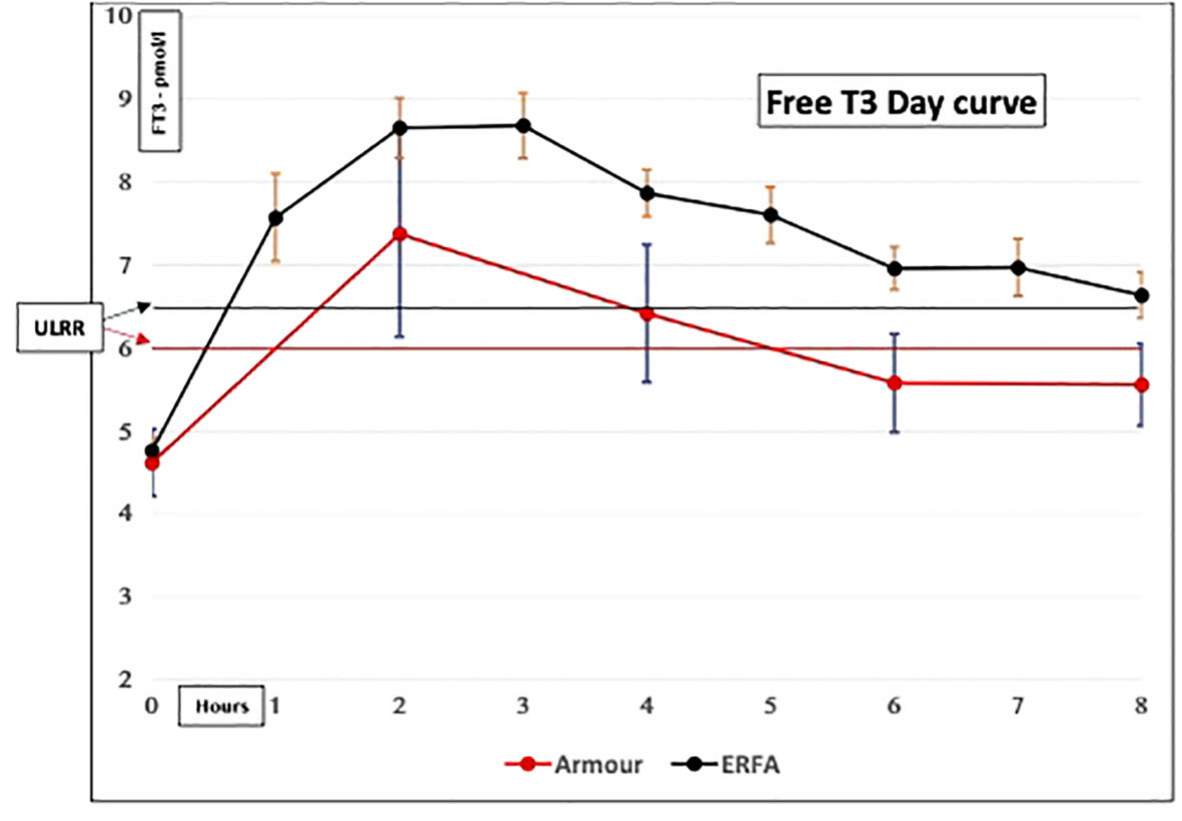
Serum-free T3 concentrations in hypothyroid subjects given natural desiccated thyroid (NDT). Both ERFA and Armour thyroid caused FT3 concentrations to be elevated above the upper limit of the reference range (ULRR) for significant amounts of time (Premawardhana L.D. et al. Oral Presentation, British Thyroid Association, May 2021).

(a) *Current short acting LT3 preparations*

(i) *Produce unphysiological “peaks”*: One reason why previous RCTs may have been inconclusive was the use of “unphysiological” LT3 preparations (21). Current LT3 preparations, including natural desiccated thyroid (NDT), are short-acting (unpublished data) (**Figures 1**, **2**). They last a few hours in the serum and produce “peaks” with persistent T3 levels above the upper limit of the reference range (ULRR) for a significant length of time, the number of peaks depending on the frequency of administration. Of the 16 RCTs for which information was available, nine used a once/day, four used a twice/day, and one used a three/day LT3 regime (**Table 1**).
(ii) *Difficult to monitor for “dose adjustment”*: The unphysiological pharmacokinetic profiles and “peaks” produced (**Figures 1**, **2**) do not lend themselves to monitoring of thyroid hormones for dose adjustment. **Figure 1** illustrates the variability of serum free T3 at different times before and after oral short-acting LT3, making recommendations about the timing of blood testing for dose adjustment impossible. The current recommendation for measuring trough free T3 levels does not consider the previously mentioned peaks and time spent outside the reference range (ULRR), which may adversely affect patients both in the short and long term. As we recently showed in hypothyroid subjects given NDT (ERFA and Armour thyroid), there was a significant difference in time spent outside the ULRR despite similar trough-free T3 concentrations (unpublished data) (**Figure 2**).

*(b) Long acting “physiological” LT3 preparations*

There have been calls from Specialist Societies to investigate long acting LT3 in RCTs in symptomatic hypothyroid subjects on LT4 (21, 47). There is a shortage of long-acting preparations with clinical utility (**Table 2**, known preparations); however, two preparations have undergone preliminary trials.

(i) Poly-zinc-liothyronine (PZL):
  PZL is a polymeric compound of zinc and LT3 that adheres to the intestinal mucosa following oral administration (61, 62). This slows down its gut transit time and in essence forms a “depot” for slow release of T3 which is then absorbed into the blood stream (63). A phase 1 trial comparing PZL and LT3 showed a 6-hour plateau in serum T3 C_max_ for PZL, which was 30% lower than that for LT3. Furthermore, T3 remained at over half of the C_max_ for more than 24 h (52). This preparation may produce more physiologically relevant T3 pharmacokinetics. However, this has not been tested in QoL studies.
(iii) Triiodothyronine sulfate (T3S): Sulfation of the hydroxyl group of T3 generates T3 sulfate (T3S), which is an inactive molecule that is naturally formed during human thyroid hormone metabolism. This targets T3 for destruction by deiodinase 1 (D1). However, if required (e.g., in hypothyroidism), T3S can be desulfated by local sulfatase enzymes (64, 65) or the gut microbiome (66) to regenerate “active” T3.

**Table 2:** Known long-acting T3 preparations and their current availability.

Preparation	Details of action if known	Current status
Oral/Gastrointestinal		
T3 granules		No information
Orodispersible films (48)	Thermal inject printing deposits LT4 and LT3 to water soluble films	Pharmacokinetics not done
T3 and swellable hydrophilic matrix (49)	T3 combined with hydrophilic swellable matrix—rate of release adjusted according to grade of Methocel and SimpleCap/Lactose matrix in capsules	First study showed lower peak T_max_ prolonged from 3.2 to 5 h (50) Second study using cellulose and Mg stearate matrix was unsuccessful (51) Current status unknown
PZL (L-thyroxine polymer with zinc)	T3 polymer with Zn–PZL. Increased gut mucoadhesion forming drug “depot” for slow release	Successful animal and Phase 1 studies (52) Awaiting regulatory approvals
T3 sulfate (T3S)	T3S desulfated by tissue and gut microbiome sulfatases providing a favorable long-acting pharmacokinetic profile	Two phase 1 human studies completed (53, 54). Phase 2b RCT funded (MR/X013170/1) and planned
Parenteral		
Aqueous and oily T3 preparations		Inconsistent results in animal studies. Current status unknown
Osmotic subcutaneous mini pumps/pellets with T3 (55)	Release T3 over many days	Animal studies successful. Current status unknown
Implantable subcutaneous min-rods with T3 (56)	Stable long term T3 release	Successful animal studies. Current status unknown
Intraperitoneal and intravenous T3 preparations (55)		Produce unphysiological T3 peaks. Current status unknown
Regenerative Methods		
Mice Stem cells expressing NKX.2 and PAX 8 grafted onto mice (57, 58)	Generates thyroid cells from stem cells leading to thyroid follicular cells when implanted in to mice	No human studies done. Current status unknown
Tissue Targeting		
T3 conjugated with glucagon (59) and the use nanotechnology to deliver T3 to the brain (60)	Conjugates of glucagon and T3 delivers to the liver	No human studies. Current status unknown

The above preparations are currently available or have being investigated.

Sulfation also confers the following qualities to T3S:

(1) Increased solubility targets biliary excretion and reabsorption in enterohepatic circulation. Sulfation and desulfation enzymes are highly expressed in the liver.
(2) T3S is markedly preferred as a substrate over T3 by the D1 deiodinase enzyme expressed in the liver, kidney, and intestine (67).
(3) D1 activity is increased in hyperthyroidism and D2 activity is reduced.

Taken together, these elements create a self-regulating system to stabilize serum T3 levels: if T3 increases, more is sulfated and excreted via the liver and kidney, and increased D1 levels at these sites result in the irreversible destruction of T3S to inactive metabolites. In contrast, in hypothyroidism, when T3 levels are low, D1 activity is low in the liver and intestine, and more T3S excreted in the bile can be reabsorbed and desulfated to restore T3 levels.

When T3S is administered orally, this system generates a favorable pharmacokinetic profile: T3S levels peak and return to baseline in 12 h, but (desulfated) serum T3 levels rise and remain high for over 48 h, likely through enterohepatic recirculation and desulfation. This result is in effect a “slow-release preparation,” as demonstrated in thyroidectomized humans in 2014 (53). Santini et al. also showed in a continuous dosing study over 11 weeks that replacing 25 μg of T4 with 40 μg of T3S in subjects on LT4 monotherapy results in a reduction in serum free T4 without a reduction in free T3. This restores a physiological T4/T3 ratio alongside a normal TSH in 89% (n = 36) of patients as compared to fewer than 45% on LT4 alone (54). A particularly attractive feature of T3S therapy is that, by exploiting natural physiology, it is self-regulating (as described above), with excess levels being rapidly destroyed and/or excreted, generating a very wide therapeutic index and level of safety. Consistent with this, no safety signals were observed in the 11-week study, which was uncontrolled and not designed or powered for clinical endpoints (54).

(3) **Appropriate trial design**

RCTs provide high-quality evidence, with minimal bias. However, they are difficult to design and execute, expensive, time-consuming, and targeted at specific groups (may therefore be difficult to extrapolate to other groups or to generalize their results).

Several broad principles have been proposed. Such a trial should be randomized (thereby matched for sex, menopausal status, age, etc.), blinded to both participants and investigators (e.g., participants’ thyroid hormone status), and placebo controlled (over-encapsulation or identical preparations). All laboratory assessments should be performed in a single reference laboratory to minimize inter- and intra-assay variations, preferably in a single batch (although this may not be practical). However, safety and thyroid tests for dose adjustment may be performed locally.

A long study duration of 12–24 months is recommended in view of the long half-life of LT4 (approximately 7 days), the time taken to achieve a “steady state” for dose adjustment, and to assess medium-term efficacy and safety for cardiac and bone tissue. However, a long trial may adversely affect enrolment, increase dropout rates, and increase cost.

Of the various trial designs available, a crossover design is attractive to participants, as they are certain to receive the intervention/drug under investigation during one of the trial phases. Furthermore, the ability to perform paired analyses enhances its statistical power, reducing the number of patients needed to be enrolled, and reducing costs. However, “carryover” of effects of one therapy to a different phase of the study, loss, and inability to analyze data if patients drop out are disadvantages. These effects can be mitigated if a parallel-group design is adopted.

## Conclusions

The issue of “optimal” treatment for hypothyroidism remains unresolved. LT4 monotherapy satisfies the majority, but a significant minority remain dissatisfied because of persistent symptoms that impair QoL. A trial of LT3 is recommended in them by international specialist societies (30, 47, 68).

Although the theoretical evidence for this recommendation is convincing, evidence for the efficacy of RCTs is limited. However, some limitations of these RCTs should be addressed in future trials: they (i) were underpowered, (ii) used inappropriate outcome measures, (iii) were mainly of short duration, and (iv) used short-acting, unphysiological LT3 preparations. We believe that the availability of long-acting LT3 preparations such as PZL and T3S, overcomes this disadvantage, and their efficacy should be investigated in phase 2b/3 RCTs.

We recommend that in such trials, researchers study subjects with a high symptom burden and propensity to respond better to combination LT3 + LT4 therapy (**Box 1**). In this regard, subjects with the D2 polymorphism (Thr92Ala) would be ideal, as they fulfilled both criteria. There is a plausible biological role of the D2 polymorphism in determining symptom load and a better response to LT3 + LT4 treatment. This strategy eliminates subjects with the potential for only a minimal, clinically insignificant response to LT3 (e.g., those with subclinical hypothyroidism).

Box 1 **Key elements of a trial of combined LT3 + LT4 therapy in hypothyroidism.**

**Aim**: To provide phase 2b/3 efficacy data that combined LT3 + LT4 therapy is superior to LT4 alone

**Primary outcome**

Symptom improvement using a thyroid specific PROM, e.g., ThyPRO39 or its composite score

**Secondary outcomes**

1. pharmacokinetic profiles of interventional drug
2. changes to weight/BMI, body composition, REE, metabolomic profiles and bone turnover.
3. safety outcomes

**Patient selection**

1. presence of symptoms and threshold for recruitment
2. use of thyroid specific PROMs
3. recognizing minimal important change in score
4. higher symptom load and greater response to LT3, e.g., deiodinase 2 snp

**LT4 dose at recruitment**

Minimum of 1.2 μg/kg/day or 100 μg/day (for pragmatic reasons)

**Cause of hypothyroidism**

Unrestricted recruitment of subjects with high symptom load and a propensity to respond to treatment

**Choice of interventional medication**

Long acting LT3 preparations e.g., PZL or T3S

**Appropriate Trial design**

Randomized placebo-controlled trial using a cross over design

PROM, patient reported outcome measures; snp, single nucleotide polymorphism.

A placebo-controlled, double-blind, crossover RCT would be ideal because of the advantages of such a design—adequately powered with limited numbers, all subjects receiving the active and placebo medications and limiting costs within acceptable limits.

## Author contributions

LP: Conceptualization, Writing – original draft, Writing – review & editing. PT: Conceptualization, Writing – review & editing. OO: Conceptualization, Writing – review & editing. MA: Conceptualization, Writing – review & editing. EO: Conceptualization, Writing – review & editing. CD: Writing – original draft, Writing – review & editing, Conceptualization.
